# Supplementary material for: Analysis of PPARγ Signaling Activity in Psoriasis
Source: Int J Mol Sci. 2021 Aug 10;22(16):8603. doi: 10.3390/ijms22168603 (PMC8395241; doi:10.3390/ijms22168603)
Supplement: Supplementary file 1 [file ijms-22-08603-s001.zip › Supplemental materials_Analysis of PPARg signaling activity in psoriasis/Pathway models/Models images and html files/PPARG inhibits phenotypes assotiated with psoriasis/244993.html]

atrial remodeling


# Disease atrial remodeling

|  |  |
| --- | --- |
| URN | urn:agi-meshdis:Atrial%20Remodeling |
| Total Entities | 0 |
| Connectivity | 505 |
| Name | atrial remodeling |

---

|  |  |
| --- | --- |
| Pathway | PPARG inhibits phenotypes assotiated with psoriasis |
|  | efects selected |

---

|  |  |
| --- | --- |
| MedScan ID | 9017534 |

---

|  |  |
| --- | --- |
| Alias | Atrium Remodeling |
|  | Atrial Remodeling |
|  | Atrial Myocardial Remodeling |
|  | Electrical Remodeling |
|  | remodeling of the atrium |
|  | Atrial Cardiac Remodeling |
|  | heart atrium remodeling |

---

|  |  |
| --- | --- |
| MeSH ID | D064752 |
|  | M0583285 |

---

|  |  |
| --- | --- |
| MeSH Heading | Atrial Remodeling |

---
